# Engineered live biotherapeutic products: design decisions, failure modes, and clinical translation

**DOI:** 10.3389/fmicb.2026.2002085

**Published:** 2026-10-16

**Authors:** Sijia Shen, Junsheng Zhao, Hainv Gao

**Affiliations:** 1 Key Laboratory of Artificial Organs and Computational Medicine of Zhejiang Province, Shulan (Hangzhou) Hospital, Shulan International Medical College, Zhejiang Shuren University, Hangzhou, China; 2 State Key Laboratory for Diagnosis and Treatment of Infectious Diseases, National Clinical Research Center for Infectious Diseases, National Medical Center for Infectious Diseases, Collaborative Innovation Center for Diagnosis and Treatment of Infectious Diseases, The First Affiliated Hospital, Zhejiang University School of Medicine, Hangzhou, China

**Keywords:** biocontainment, chassis selection, clinical translation, engineered live biotherapeutic products, genetic circuits, recombinant microorganisms, synthetic biology

## Abstract

Engineered live biotherapeutic products (LBPs) are viable microorganisms deliberately modified to perform a therapeutic function in a patient. Recombinant therapeutic strains are considered separately from conventional probiotics, donor-derived microbiota products, vaccines, living diagnostics, environmental systems, and systemically administered oncolytic bacteria. Selected oncology platforms share engineering constraints with LBPs, although their classification as LBPs depends on the specific therapy. The target product profile sets the niche, route, and exposure requirements against which chassis, genetic control, and payload release are assessed. Fitness costs, genetic stability, basal leakage, and clearance constrain circuit complexity. Formulation and manufacturing must preserve viability and mechanism-linked potency, with process control and independent containment layers evaluated quantitatively during development. Human studies of engineered metabolic strains support feasibility, short-term tolerability, clearance, and pharmacodynamic activity, although broad or durable clinical benefit has not yet been shown. Microbiota products approved by the U.S. Food and Drug Administration (FDA) provide regulatory precedents for live microbial medicines, with approval specific to each unengineered product. Clinical trials require separate assessment of target engagement and patient benefit.

## 1 Introduction

Microorganisms influence host metabolism, epithelial function, and immunity, providing opportunities for therapeutic intervention (Murali and Mansell, 2024). The U.S. Food and Drug Administration (FDA) defines a live biotherapeutic product (LBP) as a biological product containing live organisms that is intended to prevent, treat, or cure disease and is not a vaccine (FDA (U.S. Food and Drug Administration), 2016). An engineered or recombinant LBP (rLBP) is a viable microbial strain whose genome or heritable program has been deliberately modified to produce a defined therapeutic function in a patient. FDA-approved donor-derived microbiota products for recurrent *Clostridioides difficile* infection provide regulatory and manufacturing precedents for live microbial preparations, while engineered therapeutic functions require separate validation (FDA (U.S. Food and Drug Administration), 2026).

Recombinant microorganisms may combine local sensing, molecular production, and release for therapeutic delivery (Figure 1), enabling metabolic conversion or conditional delivery of short-lived effectors (Ozdemir et al., 2018; Murali and Mansell, 2024). These capabilities depend on strain, route, formulation, and host context, so evidence of colonization needs to be supplemented by measurements of delivery and efficacy. Repeated dosing may maintain activity without persistent engraftment (Charbonneau et al., 2020; Heavey et al., 2022). Conditional expression contributes to the required exposure only when the input distinguishes the target setting and basal output remains acceptable.

**FIGURE 1:**
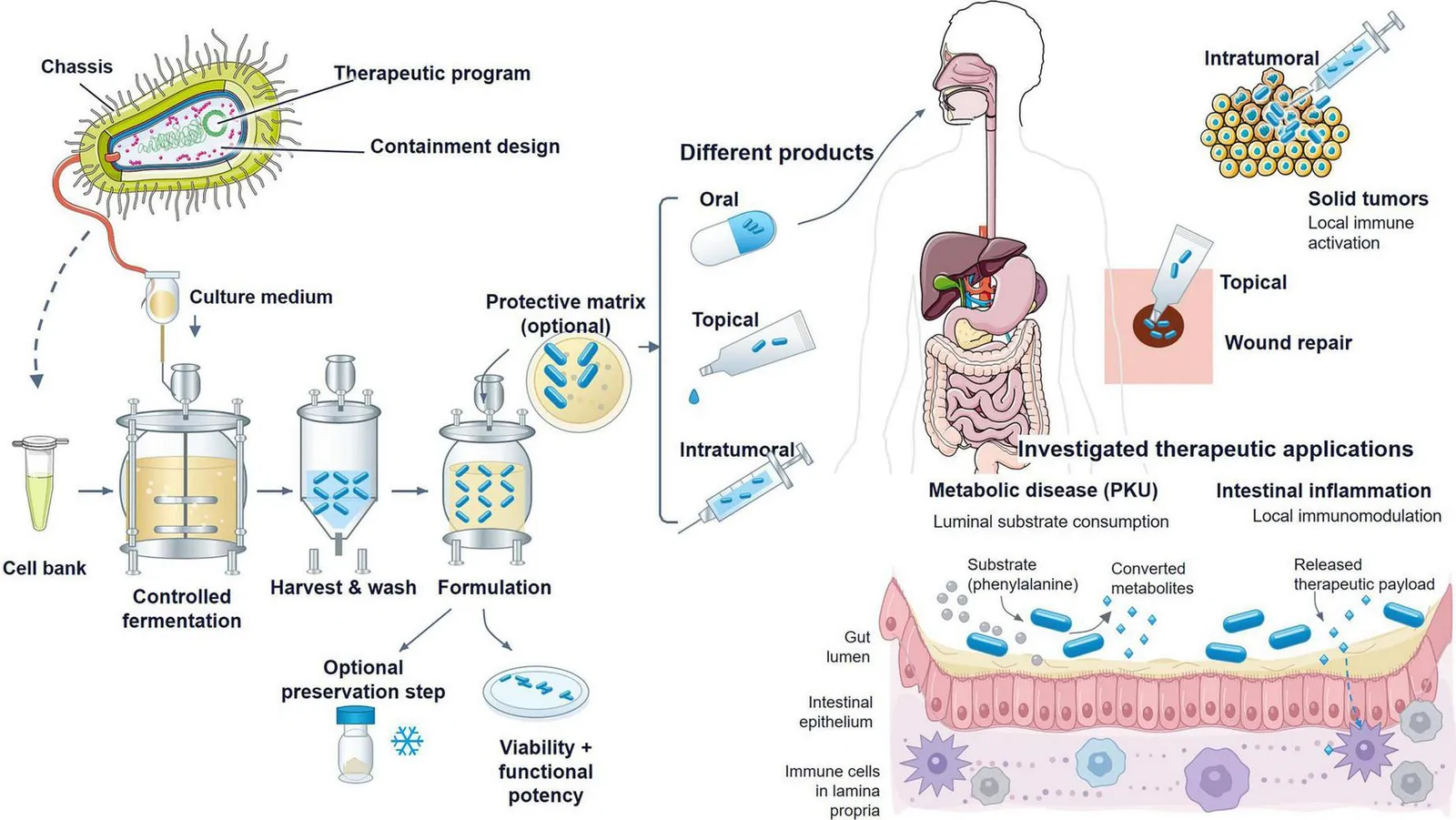
From engineered strains to therapeutic delivery. From left to right, strain design (chassis, therapeutic program and containment design) is followed by cell banking, controlled fermentation, harvest and washing, and formulation. Protective matrices and preservation steps are product-specific options, and quality assessment combines viability with functional potency (FDA (U.S. Food and Drug Administration), 2016; EDQM (European Directorate for the Quality of Medicines & HealthCare), 2018). The center shows three distinct products matched to oral, topical and intratumoral routes, respectively; each has a separate formulation. The right shows investigated therapeutic applications: luminal phenylalanine consumption in phenylketonuria (PKU) programs with early human pharmacodynamic evidence (Puurunen et al., 2021; Vockley et al., 2023), local release of therapeutic payloads in preclinical inflammatory-disease systems (Zou et al., 2025; Ding et al., 2026), topical wound repair in early human studies (Öhnstedt et al., 2023; Schindler et al., 2024), and local immune activation after intratumoral administration in an adjacent oncology platform with early human evidence (Luke et al., 2023). Blue rods and blue-filled dosage forms denote engineered bacteria, gray spheres luminal substrate, and blue diamonds converted metabolites or released payload. Whole bacteria remain in the intestinal lumen on the oral branch.

Literature selection emphasized work since 2018 while retaining earlier regulatory and clinical landmarks; clinical status was assessed against an August 2026 cutoff. Primary clinical reports, trial records, and regulator documents were prioritized for quantitative or status claims; reviews informed design principles and the selection of representative preclinical systems. Selection was non-systematic and non-exhaustive, and excluded diagnostic-only systems.

The analytical evidence tiers are E0, *in vitro* or *ex vivo* evidence; E1, small-animal proof of concept; E2, replicated evidence across mammalian species or informative large-animal preclinical evidence; E3, early human safety, feasibility, or pharmacodynamic evidence; E4, randomized human efficacy evidence; and E5, regulatory approval for a specified product and indication. A tier characterizes support for a particular claim; intrinsic construct quality is assessed separately, and an exploratory randomized study may remain E3. The tiers are not a prescribed regulatory sequence. The value of an E2 study depends on whether its model addresses a defined translational uncertainty, whereas entry into E3 requires a justified product-specific nonclinical and chemistry, manufacturing, and controls (CMC) package.

Table 1 summarizes engineering requirements and failure modes. Supplementary Table 1 records chassis, inputs, outputs, release, models, and measured organism fate, distinguishing adjacent platforms from core therapeutic LBPs. Table 2 organizes human evidence from recombinant programs, donor-derived and defined non-engineered comparators, and adjacent oncology platforms. Supplementary Table 2 groups CMC assessments of quality, functional stability, and organism fate by purpose and development stage.

**TABLE 1:** Design decisions, quantitative assessments and failure modes for recombinant live biotherapeutic products.

Decision point	Quantitative assessments	Common failure mode
Target product profile	Viable dose at release and at shelf-life end; activity per viable cell; route, compartment, onset, duration and shedding window	Viability (colony-forming units, CFU) used as the sole measure of potency
Chassis match to niche and route	Strain-level whole-genome sequencing plus orthogonal identification; survival during administration; activity at the target site; antimicrobial and phage susceptibility	Safety assigned at genus or species level
Genetic circuit design	Basal leakage; induction ratio; activation and reset kinetics; off-target cue panel in patient-derived matrices	Portability across chassis inferred from modular design
Payload and release mode	Payload identity and bioactivity; released fraction; exposure at the intended site; host-response markers	Exposure inferred from transcription or bulk culture titre
Fitness burden and evolutionary stability	Sequence and function across the maximum plausible generation count, from seed expansion to in-patient residence	Endpoint sequencing alone misses minority escape variants
Multilayer biocontainment	Escape frequency with a stated denominator and detection limit; safeguard stability; shedding; clearance; horizontal transfer	Plasmid maintenance (e.g., *hok/sok*) mislabeled as containment
Justification for consortium design	Ratio stability; exchanged metabolites; consortium versus each strain alone; potency assay capturing the division of labor	Model-specific gains interpreted as intrinsic consortium superiority
Formulation and CMC, co-developed	Mechanism-linked, stability-indicating potency assay; comparability after any change of scale, site, process or analytics	Formulation treated as a separate downstream step

Each added sensor, payload, material or strain must address a specific failure mode while retaining adequate potency, genetic stability, containment and manufacturability. CMC, chemistry, manufacturing, and controls; CFU, colony-forming units. Candidate sensing cues: oxygen, pH, inflammatory mediators, metabolites, quorum signals, temperature, and exogenous inducers.

**TABLE 2:** Human evidence for live biotherapeutic products and adjacent engineered-bacteria platforms.

Product/category	Engineered?	Construct and route	Indication and status	Design and main result	Safety and interpretation	References
REBYOTA, donor-derived fecal microbiota product; not engineered	N	Screened fecal suspension; single 150-mL rectal dose	Prevent rCDI; US approved 30 Nov 2022	Pivotal, placebo-controlled; 262 treated; Bayesian integrated 8-week success 70.6% vs 57.5%	Mainly GI reactions; residual transmissible-agent risk. Approval is specific to this donor-derived product.	FDA (U.S. Food and Drug Administration), 2022
VOWST, donor-derived spore product; not engineered	N	Screened donor-fecal spores; oral, 4 capsules/day for 3 days	Prevent rCDI; US approved 26 Apr 2023	*n* = 182 (89 active, 93 placebo); 8-week recurrence 12.4% vs 39.8%	GI/systemic reactions and residual transmissible-agent risk. Approval is specific to this donor-derived product.	FDA (U.S. Food and Drug Administration), 2023
LACTIN-V, defined non-engineered single strain	N	*L. crispatus* CTV-05; vaginal after metronidazole	Recurrent BV; phase 2b	*n* = 228 (152 active, 76 placebo); week-12 recurrence 30% vs 45%; RR 0.66 (95% CI 0.44–0.87)	Local/systemic AE rates similar.	Cohen et al., 2020
MTC01, defined non-engineered 15-strain consortium	N	Cultured same-donor isolates; colonoscopic dosing	rCDI; phase 1b	*n* = 18 (9 MTC01 vs 9 same-donor FMT); week-8 recurrence-free 7/9 vs 8/9	No treatment-related AE; safety-focused and not powered for equivalence.	Bethlehem et al., 2026
LL-Thy12, recombinant mucosal LBP	Y	Thymidine-dependent *L. lactis* secreting human IL-10; oral	Crohn’s disease; phase 1	*n* = 10, open-label; feasibility and recovery of the modified organism; no controlled efficacy estimate	Early human containment and safety findings; too small for benefit inference.	Braat et al., 2006
SYNB1020, recombinant oral LBP	Y	Engineered EcN consuming ammonia; oral	Hyperammonemia program; phase 1	Healthy-volunteer dose escalation; dose-dependent exposure and pathway activity	Patient efficacy remains unestablished.	Kurtz et al., 2019
SYNB1618, recombinant oral LBP	Y	Engineered EcN phenylalanine catabolism + auxotrophy; oral	PKU; phase 1/2a	*n* = 70 (56 healthy, 14 PKU); randomized placebo-controlled components; dose-responsive pathway metabolites	Mostly mild/moderate GI AE; cleared within 4 days; no efficacy conclusion.	Puurunen et al., 2021
SYNB1934, recombinant oral LBP	Y	Enhanced phenylalanine-degrading EcN; oral	PKU; phase 2; pivotal program discontinued	Phase 2 *n* = 20, 15 completed, open-label; D5-Phe AUC −43%, fasting Phe −40%; pivotal endpoint later predicted futile	No serious infection; four AE discontinuations. Clinical efficacy remains unconfirmed.	Vockley et al., 2023; ClinicalTrials.gov, 2023
AG019, recombinant oral LBP	Y	Engineered *L. lactis* delivering proinsulin + IL-10; oral ± teplizumab	Recent-onset type 1 diabetes; phase 1b/2a	42 analyzed (24 monotherapy, 18 combination); primary safety objective supported; immune and metabolic findings exploratory	No serious AE, product-related discontinuation, or systemic organism/product exposure.	Mathieu et al., 2024
SYNB1891, adjacent intratumoral engineered bacterium	Y*	EcN producing STING agonist; intratumoral ± atezolizumab	Advanced malignancies; phase 1; business termination	*n* = 32 (24 monotherapy, 8 combination); target engagement; stable disease in 4; no controlled efficacy estimate	Five CRS events, one DLT; no related infection. Adjacent intratumoral oncology platform, outside the conventional mucosal LBP category.	Luke et al., 2023; ClinicalTrials.gov, 2019
VNP20009, adjacent systemic tumor-targeting bacterium	Y*	*S.* Typhimurium Δ*purI*/Δ*msbB*; intravenous	Metastatic melanoma/renal cancer; phase 1	*n* = 25, uncontrolled; tumor colonization in 3; no objective regression	Bacteremia/multisystem toxicity at higher doses; murine tropism did not translate.	Toso et al., 2002
*C. novyi*-NT, adjacent oncolytic bacterium	Y*	Alpha-toxin-deleted spores; intratumoral	Refractory solid tumors; phase 1	*n* = 24, uncontrolled; germination or lysis in 10/24; injected-tumor reduction in 9/22 evaluable	Grade 4 sepsis *n* = 2; gas gangrene *n* = 1. Outside conventional LBP guidance.	Janku et al., 2021

Approval applies only to the stated jurisdiction and indication. Engineered? Y, recombinant organism; N, not genetically engineered; Y*, engineered organism used in an adjacent oncology platform. The donor-derived approvals are product-specific and provide no approval for recombinant LBPs. AE, adverse event; AUC, area under the curve; BV, bacterial vaginosis; CI, confidence interval; CRS, cytokine-release syndrome; D5-Phe, deuterium-labeled phenylalanine; DLT, dose-limiting toxicity; EcN, *Escherichia coli* Nissle 1917; FMT, fecal microbiota transplantation; GI, gastrointestinal; IL-10, interleukin-10; LBP, live biotherapeutic product; Phe, phenylalanine; PKU, phenylketonuria; rCDI, recurrent *Clostridioides difficile* infection; rLBP, recombinant LBP; RR, risk ratio; STING, stimulator of interferon genes.

## 2 Engineering design of recombinant live biotherapeutic products

### 2.1 Target product profile

A target product profile specifies indication, patient population, route, target compartment, onset and duration, dosing frequency, co-medications, and acceptable shedding. These choices define the required exposure and provide a basis for selecting the strain and genetic architecture. A design for transient luminal metabolism may be unsuitable when tissue penetration or systemic protein exposure is required. Clinical studies of metabolically active engineered strains have demonstrated pharmacodynamic activity during dosing followed by organism clearance, supporting an exposure model based on transient activity without permanent engraftment (Kurtz et al., 2019; Puurunen et al., 2021).

The target product profile can be translated into acceptance criteria for viable dose, activity per viable cell, basal and induced output, response time, and performance in relevant biological matrices. For a strain intended to remove a toxic metabolite, cell count measures viable dose, whereas substrate consumption or product formation under physiologically relevant conditions can provide a mechanism-linked potency measure (FDA (U.S. Food and Drug Administration), 2016; Charbonneau et al., 2020).

### 2.2 Chassis selection for niche, route, and developability

Chassis choice constrains survival during administration, activity in the target compartment, payload burden, interactions with the microbiota and immune system, susceptibility profiles, manufacturability, and prior human exposure (Murali and Mansell, 2024; Byun et al., 2026). Candidate assessment can combine whole-genome analysis with strain-level phenotyping for virulence, toxigenicity, antimicrobial resistance, translocation potential, and unintended metabolic products. Favorable health associations reported for an unmodified commensal motivate candidate selection; safety and therapeutic activity require testing in its engineered derivative.

Established probiotic chassis offer human-use experience and accessible production methods, but they may be difficult to engineer, may express weakly *in vivo*, and often persist inconsistently. Human-derived next-generation commensals may fit a particular nutrient or spatial niche more closely, yet they can impose anaerobic manufacturing, genetic-tool, stability, or safety challenges. Consensus discussions support systematic genotypic, phenotypic, functional, and microbiological safety assessment of candidate LBPs. These recommendations mainly address unmodified single strains, so their application to recombinant products requires validation of the engineered strain (Tseng et al., 2025).

Chassis comparisons depend on the indication and route. For gastrointestinal products, the relevant properties are viability and function at the luminal or mucosal site over the required interval. Assessment of skin and vaginal products concerns barrier interaction, local inflammation, community disruption, and formulation compatibility. Tumor-associated *Salmonella*, *Escherichia*, *Listeria*, and obligate anaerobes require a separate route-specific benefit–risk assessment (Lee et al., 2025).

### 2.3 Genetic circuits: input–processing–output–release functions

A genetic circuit is an engineered arrangement of regulatory deoxyribonucleic acid (DNA) and interacting gene products that controls cellular behavior or therapeutic output (Ozdemir et al., 2018). Input sensing, signal processing or control logic, therapeutic output, and release provide separable points for measuring circuit function; constitutive designs need not contain an inducible sensor. Measurements of sensing, expression, protein activity, and release help localize failure before attributing it to payload action at the host target. Containment governs organism survival and should remain independently testable.

Constitutive expression may be appropriate when the tolerated exposure window is wide, local substrate availability governs activity, or dosing provides sufficient control. Conditional expression is justified when spatial or temporal restriction improves the intended exposure profile. Candidate inputs include oxygen, pH, inflammatory mediators, metabolites, quorum signals, temperature, and externally supplied inducers (Ozdemir et al., 2018). Assessing whether these physiological cues discriminate the intended clinical setting calls for testing clinically relevant cue ranges, off-target conditions, basal leakage, and activation and reset kinetics in heterogeneous biological samples.

Logic gates, feedback, quorum sensing, and synchronized lysis can coordinate expression, but their performance depends on the chassis and physiological setting (Aggarwal et al., 2020). Programming *Bacteroides thetaiotaomicron* in the murine gut required chassis-specific promoters, inducible systems, and memory modules (Mimee et al., 2015). Cell-free assays, computational models, and multi-omics analyses can help prioritize designs, followed by comparison with a defined baseline in the intended organism and biological context to test any predicted improvement.

Cells produce and release autoinducers whose local concentrations reflect both cell abundance and the effects of production, diffusion, flow-mediated removal, and spatial organization (Kirisits et al., 2007; Darch et al., 2018). Matrix retention can also limit signal escape, as shown for a homoserine lactone in *Pseudomonas aeruginosa* biofilms (Li et al., 2022). Autoinducer detection then changes bacterial gene expression; therapeutic release and host responses are downstream events. In quorum-controlled lysis, the relevance of a density threshold measured in well-mixed culture to an *in vivo* safety boundary needs to be evaluated through activation and release measurements across the relevant transport and matrix conditions.

### 2.4 Payload pharmacology and release mode

Engineered LBPs can transform host or dietary metabolites, consume toxic substrates, produce small molecules, or express enzymes, peptides, binding proteins, cytokines, and antimicrobials (Murali and Mansell, 2024). Payload selection depends on the required compartment and exposure. For intestinal delivery, a luminal enzyme may act without epithelial uptake, whereas a peptide intended for systemic action requires transport across the epithelial barrier. Evaluation of local production includes both its potential to reduce degradation before the target site and measurements of absorption, bioavailability, dose consistency, and performance relative to a conventional formulation.

Release architecture determines which fraction of a produced payload can reach its target. Active secretion, programmed lysis, and surface display have different implications for accessibility, viable-cell number, and exposure to microbial inflammatory components (Chawla et al., 2026). Payload identity and bioactivity, released fraction, target-site exposure, and host response characterize release performance; organism fate requires independent measurement. Synchronized lysis generated pulsatile cargo release while a surviving subpopulation reseeded successive cycles (Din et al., 2016). A modified type III secretion platform delivered functional nanobodies (small antibody-derived binding proteins) from engineered *Escherichia coli* in the murine gut (Lynch et al., 2023).

### 2.5 Fitness burden and evolutionary stability

Therapeutic expression can impose a fitness cost, allowing non-producing cells to outgrow producers through plasmid loss or changes in the payload and its regulation (Leeflang et al., 2025). Paired sequence and functional measurements, with sampling able to detect minority escape variants, would assess whether identity, output, and safeguard function remain within specification across the plausible generation count during production and residence in the patient.

Candidate strategies include chromosomal integration, lower-burden expression, balanced pathway flux, marker-free selection, and coupling product maintenance to fitness, with direct comparison required in the chosen chassis (Charbonneau et al., 2020). In the tested bioprocess, synthetic product addiction prolonged mevalonic-acid production across 95 generations by coupling output to essential functions (Rugbjerg et al., 2018). Environmental containment and control of persistence in a patient require data on those endpoints.

### 2.6 Multilayer biocontainment

Organism persistence and engineered-DNA dissemination are separate endpoints for containment assessment. Auxotrophy (dependence on an externally supplied nutrient), conditional replication, kill switches, synthetic nutrient dependence, and physical barriers can provide complementary safeguards, with each contribution assessed through its measured function (Ozdemir et al., 2018). Selection of multiple layers begins with identifying plausible rescue conditions and shared failure mechanisms.

Toxin–antitoxin systems such as *hok/sok* favor plasmid-bearing cells, a plasmid-maintenance function distinct from organismal or genetic containment. Effects on shedding, horizontal transfer, and survival outside the intended setting require direct assessment (Ozdemir et al., 2018). Retained antibiotic susceptibility supports a potential clinical rescue strategy, while intrinsic regulation of bacterial population size is a separate engineering function. Establishing complete clearance requires assessment under the relevant exposure and tissue conditions in addition to *in vitro* susceptibility testing.

Safeguard comparisons require stated escape denominators and detection limits, including assessment of conditions that could rescue growth or impair killing. In studies of clustered regularly interspaced short palindromic repeats (CRISPR)-based kill switches in *Escherichia coli* Nissle 1917 (EcN), investigators combined genetic-stability testing with recovery from the murine gut and after excretion, and found that ecological competition and escape recovery affected apparent control (Rottinghaus et al., 2022).

### 2.7 Division of labor in microbial consortia

Multi-strain products can distribute incompatible pathways or provide complementary substrate use through cross-feeding (Jennings and Clavel, 2024; Qiao et al., 2025). Their evaluation must also address changing strain ratios, competitive exclusion, and substitution of exchanged metabolites by resident organisms. Signaling molecules used by one engineered strain may activate a receptor or regulatory module in another. Evaluation of this potential cross-reaction, together with strain-specific measurements and a community-level functional endpoint, is needed to attribute potency.

Consortium selection requires a demonstrated advantage over a single strain under relevant conditions. Comparisons include each component alone, ratio stability, exchanged metabolites, and perturbation by the relevant microbiota or diet. Animal-model improvements support model-specific conclusions; resilience and clinical superiority require evidence in the corresponding settings.

### 2.8 Formulation and manufacturing

Formulation and manufacturing should be tested before the genetic construct is fixed, since processing, storage, reconstitution, and administration can affect both viability and the functional output of the engineered genetic program (Heavey et al., 2022). Assessment of encapsulation should identify its contribution to a defined delivery barrier through comparison with the unformulated strain using the same mechanism-linked potency endpoint. For constructs validated in mice, progression to a clinical product depends on resolving the exposure and manufacturing uncertainties identified by those experiments.

## 3 Engineered bacterial platforms in oncology: an adjacent translational test case

### 3.1 Scope and regulatory boundary

Much of the bacterial-oncology literature concerns intravenous or intratumoral attenuated organisms, oncolytic bacteria, microbial vaccine vectors, or bacteria–material hybrids. These platforms share engineering tools with recombinant LBPs but differ in route, mechanism, systemic exposure, acceptable toxicity, and regulatory pathway. The FDA LBP guidance explicitly excludes oncolytic bacteria and microbial gene-therapy vectors (FDA (U.S. Food and Drug Administration), 2016).

### 3.2 From tumor access to therapeutic effect

Bacterial antitumor activity depends on survival after administration, tumor access, maintenance of genetic function, payload production and release, and host target engagement. Hypoxia, necrosis, abnormal vasculature, and immune suppression can favor selected bacteria in animal tumors, but these features vary across cancers (Lee et al., 2025). Time-resolved, strain- and model-specific tumor-to-organ recovery provides a measure of tumor selectivity.

In phase 1, intravenous attenuated *Salmonella* VNP20009 produced documented tumor colonization in 3 of 25 participants and no objective responses (Toso et al., 2002). Intratumoral *Clostridium novyi*-NT achieved germination or lysis in 10 of 24 participants and reduction of the injected tumor in 9 of 22 evaluable participants. Grade 4 sepsis occurred in two participants and gas gangrene in one after local administration (Janku et al., 2021). These studies provide E3 evidence, with randomized efficacy still to be established. Progression to an E4 trial of patient benefit depends on linking tolerated dosing and measured tumor exposure to pharmacodynamic engagement in humans (Sieow et al., 2021).

## 4 Engineered live biotherapeutics for inflammatory, metabolic, and infectious diseases

### 4.1 Intestinal inflammation

For oral inflammatory bowel disease (IBD) therapy, a dual-key EcN construct deleted asd and thyA, genes needed for essential biosynthetic functions, and supplied their functions on two complementing plasmids. Its LuxI/LuxR system produced and detected an autoinducer, whose local accumulation activated expression of the bacteriophage lysis protein φX174E. Lysis released an interleukin-2 (IL-2) variant biased toward regulatory T (Treg) cells and Amuc_1100, an *Akkermansia muciniphila* outer-membrane protein (Figure 2). The combined construct improved immune, barrier, and inflammatory readouts in murine colitis (E1; Ding et al., 2026). Replication in a complementary disease model and a physiologically relevant second species, with simultaneous measurement of local IL-2 exposure and nutrient rescue of the auxotrophies, would extend this evidence toward E2.

**FIGURE 2:**
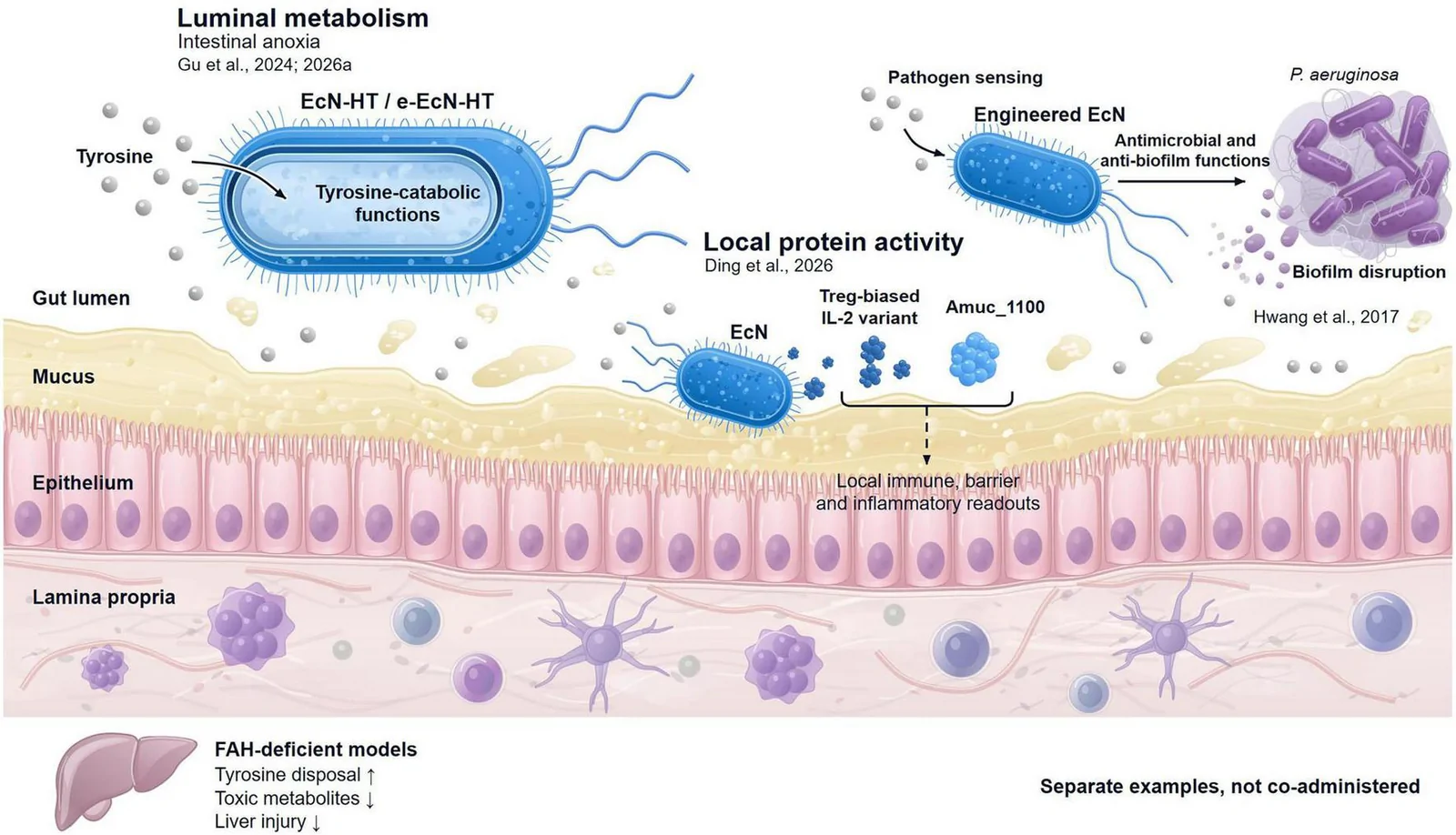
Spatial modes of therapeutic action in the intestine. A shared intestinal cross-section (gut lumen, mucus, epithelium, lamina propria) compares three engineered-bacterial strategies evaluated separately in preclinical studies. Under luminal metabolism, the *Escherichia coli* Nissle 1917 (EcN) derivatives EcN-HT and e-EcN-HT perform tyrosine-catabolic functions under intestinal anoxia, with metabolic and liver-protection outcomes reported in fumarylacetoacetate hydrolase (FAH)-deficient models of hereditary tyrosinemia type 1 (mice and rabbits) and oral labeled-tyrosine metabolism evidence in minipigs (Gu et al., 2024, 2026a). Under local protein activity, a separate EcN construct releases a regulatory T (Treg)-cell-biased interleukin-2 (IL-2) variant and Amuc_1100. The dashed arrow links their collective activity to local immune, barrier and inflammatory readouts in murine colitis; attribution to either protein and the transport mechanism remain unspecified (Ding et al., 2026). In the third strategy, engineered EcN senses *Pseudomonas aeruginosa* and deploys antimicrobial and anti-biofilm functions in animal gut-infection models (Hwang et al., 2017). Solid arrows indicate the supported functional relationships at the level examined; receptor pathways remain unspecified. Whole bacteria remain on the luminal side of the epithelium, and the liver denotes the site of the measured outcome.

A mucus-coated system placed an engineered EcN sensor–effector strain inside polyserine-modified alginate microspheres and added a hyaluronic-acid/epigallocatechin-gallate layer (Zou et al., 2025). The material improved gastric survival and intestinal adhesion or detectable residence for up to 24 h while permitting biomarker entry and output of the immunomodulatory protein AvCystatin; the combined system improved mouse IBD readouts (E1). The evidence concerns transient retention; durable colonization remains unestablished. The exposure–response relationship under different intestinal transit and mucus conditions could be tested for reproducibility in a relevant second species where informative (E2 objective). Unencapsulated bacteria, empty material, and sensor-disabled controls, assessed alongside viable counts and local protein exposure, would separate material protection from biomarker-dependent treatment.

### 4.2 Gut–brain delivery

EcN-GadABC@Fe-NE combined a temperature-responsive *gadABC* module for gamma-aminobutyric acid (GABA) production with Fe_3_O_4_ nanoparticles that converted an alternating magnetic field (AMF) into heat; a poly-norepinephrine layer improved oral survival and retention (Wang et al., 2025). AMF induced GABA release, and chemogenetic reactivation of gastrointestinal vagal afferents attenuated the behavioral effect. The construct reduced anxiety-like behavior in restraint-stressed mice (E1). Validation toward E2 would examine whether the GABA exposure–vagal response relationship is reproducible in an additional relevant species, with intestinal thermal dosimetry and nanoparticle-fate measurements at the intended field settings. Human exposure and target-engagement endpoints for a defined indication would then be needed in an E3 study. Benefit in other neurological disorders remains unestablished by these mouse data.

### 4.3 Metabolic disease

For hereditary tyrosinemia type 1 (HT1), EcN-HT expressed tyrosine-catabolic functions under intestinal anoxia; oral dosing increased tyrosine disposal, reduced toxic metabolites, and protected mice deficient in fumarylacetoacetate hydrolase (FAH) from liver injury (E1; Gu et al., 2024). In subsequent studies of the optimized e-EcN-HT construct, activity was reported in FAH-deficient mice and rabbits, and metabolism of oral ^13^C-tyrosine was demonstrated in minipigs, without detected systemic dissemination or major microbiome disruption in the reported studies (Gu et al., 2026a). This multi-species evidence reaches E2; clinical evidence remains absent. For an E3 study, the pharmacological priorities are tyrosine mass balance, exposure to L-3,4-dihydroxyphenylalanine (L-DOPA) and other products, effects of diet and nitisinone treatment, and reversibility after dosing stops.

A *Bacteroides xylanisolvens*–*Clostridium butyricum* combination used reciprocal folate and para-aminobenzoate exchange to improve ecological stability and metabolic outcomes in high-fat-diet mice (Qiao et al., 2025). Both strains were non-engineered, making this E1 study an adjacent ecological comparator. Replication of strain coexistence and metabolic output across diets and a second host species would provide a basis for progression toward E2. Application to an engineered strain requires separate validation.

### 4.4 Infectious and vaginal indications

An engineered EcN strain sensed *Pseudomonas aeruginosa* and deployed antimicrobial and anti-biofilm functions in nematode and mouse gut-infection models (E1; Hwang et al., 2017). Progression toward E2 could test pathogen suppression across clinically relevant isolates and an additional mammalian host context, together with resistance emergence and effects on commensal communities. Subsequent E3 evaluation would need to establish sensor activation at human intestinal pathogen burdens and local tolerability of the antimicrobial output.

In a randomized phase 1b study of recurrent *Clostridioides difficile* infection (rCDI), 7/9 recipients of the manufactured non-engineered 15-strain product MTC01 and 8/9 recipients of same-donor fecal microbiota transplantation (FMT) remained recurrence-free at 8 weeks. Engraftment was durable, and no treatment-related adverse events were reported across the small groups (Bethlehem et al., 2026). With 18 participants and safety as the primary objective, the study provides E3 evidence; equivalence or non-inferiority to FMT remains unestablished.

The non-engineered product LACTIN-V contains *Lactobacillus crispatus* CTV-05 and was administered intravaginally after metronidazole to restore a protective vaginal community. In the phase 2b trial, bacterial vaginosis (BV) recurrence by week 12 was 30% with LACTIN-V and 45% with placebo (E4; Cohen et al., 2020). A separate multi-omics analysis found an *L. crispatus*-dominant microbiota, an engraftment endpoint, in 30% versus 9% and identified baseline ecological predictors of colonization (Bloom et al., 2026).

## 5 Delivery systems and biohybrid materials for engineered bacterial therapeutics

Assessment of formulation-dependent changes in transit, retention, and biodistribution requires measurements of functional payload exposure at the intended site (Heavey et al., 2022). Simulated-fluid protection and tumor fluorescence are intermediate readouts. Comparisons with unformulated bacteria, empty material, non-functional strains, and free payload where applicable would help relate viable counts, payload exposure, and pharmacodynamics to disease outcomes.

### 5.1 Oral protection and intestinal retention

In the IBD microsphere system, the coating preserved biomarker-responsive AvCystatin output and improved mouse disease readouts (E1; Zou et al., 2025). Formulation development would need to establish whether local payload exposure is preserved across changes in particle size, release, food intake, and intestinal transit.

A vaccine-delivery system used a programmable oral hydrogel with chitosan–alginate capsules to protect engineered bacteria, dissolve in the intestine, and expose a synchronous lysis circuit that periodically released a nanovaccine (Zhang et al., 2023). Oral treatment inhibited tumor volume by approximately 75% and increased T-cell responses in mice bearing subcutaneous tumors (E1). The response was consistent with gut-mediated systemic immunization; bacterial delivery to the tumor was neither required nor demonstrated. Extension toward E2 could test the reproducibility of antigen exposure and immune engagement in an additional relevant species, with conventional vaccination as a comparator and repeated-lysis tolerability as an accompanying measure. Differences in models, endpoints, and routes preclude ranking this inhibition value against the light-gated EcN-pDawn-φX174E/TRAIL result.

Gelatin methacryloyl (GelMA) microgels containing bacteria that produced C–C motif chemokine ligand 21 (CCL21) extended intestinal residence and suppressed an orthotopic colon tumor after oral gavage (Chen et al., 2024). “Injectable” described the material properties; the administered route was oral. Local CCL21 exposure and immune recruitment would need replication in an additional relevant host model with a clinically usable induction method for E2 assessment of this E1 system. Matched retention controls and measurements of protein release from intact bacteria would test whether prolonged residence mediates the treatment effect.

### 5.2 Parenteral depots

Hydrogel microcapsules containing bacteria with a production–lysis circuit created a subcutaneous protein depot (Han et al., 2022). In diabetic rats, the glucagon-like peptide-1 receptor agonist exendin-4 was released for about 2 weeks and reduced glucose; a vaccine configuration induced specific immunity in mice. These distinct payload–indication combinations each provide E1 evidence. For the diabetes product, an E2 study could evaluate sustained, reproducible exendin-4 exposure and glucose control in an additional relevant species against a conventional long-acting formulation. Route-specific safety assessment would address depot retrieval or termination, capsule integrity, and escape of viable cells.

### 5.3 Tumor-targeting biohybrids

Surface attachment of sorafenib lipid nanoparticles to non-recombinant *Bifidobacterium longum* increased tumor nanomedicine accumulation by 66% in a mouse hepatocellular carcinoma (HCC) model and enhanced immune readouts under ultrasound (E1; Xu et al., 2025). Reproducible tumor-to-organ drug exposure in another relevant species would support progression toward E2. Tyrosinase-engineered *Escherichia coli* MG1655 produced melanin for tumor-associated photoacoustic imaging and photothermal therapy and suppressed 4T1 tumors after irradiation (Song et al., 2024). E2 validation of this E1 construct would examine the coupling between melanin output and therapeutic thermal dose at greater tissue depth, while quantifying heating of non-target organs.

GDOX@HSEc coupled bacterial heparan sulfate 6-O-endosulfatase 1 expression with surface-bound doxorubicin glycogen nanoparticles, whereas HRB@LC paired hypoxia-gated anti-CD47 antibody production with liposomes carrying cyclic guanosine monophosphate–adenosine monophosphate (cGAMP), an innate immune agonist (Yang et al., 2023; Dong et al., 2026). Both improved mouse tumor outcomes (E1). GDOX@HSEc enzyme activity and drug release would need to be related reproducibly to organ exposure in a second relevant species (E2 objective). Concordant antibody and cGAMP target engagement at a tolerable exposure would be the corresponding E2 requirement for HRB@LC. Component-matched controls would allow evaluation of synergy and reduced systemic toxicity for either combination.

EcN conjugated to AS1411, a tumor-targeting nucleic-acid aptamer, and expressing C–X–C motif chemokine ligand 9 (CXCL9) increased tumor accumulation after tail-vein dosing, recruited CD8^+^ T cells, and suppressed Lewis lung tumors in mice (Gu et al., 2026b). Antibiotics lowered peak bacterial burden while leaving a viable tumor reservoir. For this E1 platform, validation toward E2 could examine the reproducibility of tumor-to-organ bacterial and CXCL9 exposure in a second relevant species, including the residual reservoir after antibiotic treatment.

Deletion of *nlpI*, encoding an outer-membrane lipoprotein, increased outer-membrane vesicle (OMV) production in a live engineered EcN construct designated ENHL. Following oral dosing, arabinose induced bacterial lactate oxidase (LOx) expression, and LOx-bearing vesicles provided an additional route for enzyme delivery. The study reported tumor bacterial counts, LOx protein measurements, lactate depletion, and improved radiotherapy outcomes in a mouse colorectal-cancer model (E1; Peng et al., 2026). The relative transport and therapeutic contributions of live cells and vesicles remain incompletely resolved. Further work toward E2 could combine replication in an additional relevant host model with time-resolved measurements of inducer, bacterial, vesicle, and enzyme exposure, including effects on lactate outside the tumor.

Structural heterogeneity has been demonstrated in vesicles from a hypervesiculating EcN tolR mutant (Pérez-Cruz et al., 2016). OMVs also contain lipopolysaccharide (LPS) and membrane proteins; experiments with *P. aeruginosa* vesicles demonstrated that both components contributed to macrophage inflammatory responses (Ellis et al., 2010). Carrier uniformity and intrinsic immune effects remain unresolved for ENHL vesicles. Payload-null vesicles and matched LPS/protein measurements would help separate LOx-dependent activity from the carrier’s immune contribution.

Material rupture, cargo detachment, diffusion, and particle persistence need evaluation in the relevant compartment. Environmental living materials and *ex vivo* production hosts offer design analogies; their application to therapeutic delivery requires independent validation (Lin et al., 2024).

## 6 Stimuli-responsive and spatiotemporally controlled engineered bacteria

Organism localization, stimulus delivery or sensing, bacterial gene expression and release, and host target engagement are distinct steps in stimulus-controlled therapy. Exogenous light, ultrasound, and magnetic fields provide operator-defined timing; hypoxia, acidity, and lactate provide disease-associated but non-exclusive cues. Quorum sensing responds to local autoinducer accumulation, which depends jointly on bacterial abundance and signal transport through the surrounding matrix (Darch et al., 2018). Evidence for these approaches remains preclinical, leaving human control of spatial and temporal exposure unvalidated.

### 6.1 Light-gated expression and lysis

EcN-pDawn-φX174E/TRAIL used the blue-light-responsive pDawn system to drive bacterial lysis through φX174E and release tumor necrosis factor-related apoptosis-inducing ligand (TRAIL) after tumor accumulation (Zhang et al., 2022). Tumor-growth inhibition in a mouse colorectal model was 53% with light versus 11.8% without it (E1). The contributions of illumination, bacterial viability, and inflammation were not fully separated by this comparison. Further E2 validation would examine the reproducibility of the light–lysis–payload exposure relationship in anatomically relevant tissue, including a second species where needed to assess penetration. Superficial or endoscopically accessible lesions offer a plausible initial setting. Evaluation of repeated dosing would require light-only and payload-null controls.

In the intravenously administered EcN-*flaB*–upconversion nanoparticle (UCNP) system, UCNPs converted 808-nm near-infrared (NIR) irradiation to blue light. This activated the bacterial EL222/pC120 light-response system and induced flagellin B production. The payload then engaged Toll-like receptor 5 (TLR5) on host cells, with downstream signaling involving myeloid differentiation primary response 88 (MyD88); tumor inhibition was reported in mice (E1; Zhu et al., 2023). A separate platform, NIR-nanoantenna-sensitized engineered bacteria (NASEB), coated MG1655-pDawn-HlyE with UCNPs; local light conversion induced the pore-forming protein hemolysin E (HlyE) (Tao et al., 2023). For both E1 platforms, progression toward E2 depends on quantitative confirmation of the conversion–expression–host-response sequence at clinically relevant tissue depths in a suitable second species, with matched thermal controls and measurement of UCNP fate.

### 6.2 Ultrasound and magnetic fields

The MG1655 construct Ec@DIG-GVs produced gas vesicles for ultrasound imaging and interferon-γ (IFN-γ) from a heat-responsive genetic circuit, while carrying doxorubicin through an acid-labile linkage (Yang et al., 2024). After tumor localization, focused-ultrasound heating induced bacterial IFN-γ production; acidic conditions separately released doxorubicin. The combined treatment improved immune and tumor outcomes in mice (E1). The relationship between delivered thermal dose, IFN-γ exposure, and drug release could be tested for reproducibility in a relevant second species (E2 objective). Abedi et al. (2022) used a temperature-actuated state switch in intravenously administered EcN to release immune-checkpoint nanobodies, small antibody-derived binding proteins, after focused-ultrasound hyperthermia (E1). Switching reproducibility and payload exposure under human-scale perfusion, motion, and acoustic conditions would be the E2 endpoints for this construct. Both systems require calibration of the device output against the bacterial response (Wang et al., 2026).

The oral EcN-GadABC@Fe-NE system converted AMF exposure into heat to induce GABA production in restraint-stressed mice (E1; Wang et al., 2025). Spatial mapping of applied field, intestinal temperature, and GABA output would assess control performance by separating field penetration from selective activation.

### 6.3 Multi-input and endogenous control

An oral EcN colorectal-cancer platform used lactate to activate cytolysin A (ClyA) production and NIR photothermal signaling to induce the membrane-active peptide melittin, bacterial lysis, and ClyA release (Chen et al., 2025). Treatment improved mouse outcomes (E1). Validation toward E2 could examine the reproducibility of payload exposure across tissue lactate and thermal conditions in an additional relevant species. The design is consistent with sequential control of production and release; classification as a strict AND gate requires evidence of its joint input–output behavior. For this analysis, the probability of both inputs activating erroneously in a specified non-target tissue equals the probability of error in the first input multiplied by the conditional probability of error in the second, given the first. This reduces to the product of their separate error probabilities only when the input errors are independent. Estimating clinical risk additionally requires measured input-error rates, dependence, and output leakage. A tissue-level input–output table and comparisons with each single-input construct would test the proposed safety advantage.

PD-1@EcM used an acid-responsive promoter in MG1655 to produce a mouse antibody single-chain variable fragment (scFv) against programmed cell death protein 1 (PD-1) after intravenous accumulation in residual HCC (Li et al., 2025). A single post-ablation dose increased CD8^+^ responses, shifted macrophage phenotypes, inhibited primary and metastatic tumors, and prolonged survival in mice (E1). Extracellular scFv exposure and checkpoint engagement could be tested for reproducibility across residual-tumor acidity conditions in another relevant species, using a species-compatible payload (E2 objective). Because acidity also occurs outside tumors, any claim of fewer immune-related adverse events would require matched comparisons of systemic exposure and toxicity.

In the dual-key EcN colitis system, LuxI-generated autoinducer accumulated locally, engaged LuxR, and activated φX174E-mediated bacterial lysis, releasing the therapeutic proteins (Ding et al., 2026). Signal-dependent lysis controls bacterial output; therapeutic need remains a separate variable. Signal accumulation reflects production, diffusion, washout, and matrix-dependent transport; studies of *P. aeruginosa* aggregates demonstrate that spatial organization and mucin can alter quorum communication (Darch et al., 2018). To extend this E1 evidence toward E2, the autoinducer-to-lysis transfer function would need replication under relevant intestinal flow and mucus conditions in another host species. Quantifying possible matrix binding and signal degradation would establish their contribution to control performance.

### 6.4 Comparing modalities and translating control

Light-based systems require evidence that the delivered optical signal reaches the bacteria; thermal systems require calibration of local temperature against gene expression; endogenous sensing requires evaluation across overlapping diseased and healthy tissue cues; and quorum control requires local signal-transport measurements. Activation threshold, on/off ratio, response and decay times, payload exposure, repeatability, and off-target activation provide a basis for comparison within the relevant tissue context (Chang et al., 2025).

Payload switching and organism persistence require separate assessments. Validation of switching would include challenges to the off state in healthy tissues and under plausible confounders, such as inflammation, ischemia, and unrelated device exposure, selected for the chosen input (Ozdemir et al., 2018).

For products combining bacteria, material, drug cargo, and an activating device, stimulus control requires a reproducible relationship between the specified input and functional output. Sensor-disabled and output-null strains, stimulus-only and unloaded-material arms, and free therapeutic where applicable can help assign component contributions. Improved precision or safety requires measured gains in target-site function accompanied by lower non-target exposure or toxicity.

## 7 Clinical translation, CMC, regulation, and responsible development

### 7.1 Product taxonomy and clinical evidence

Donor-derived microbiota products, defined non-engineered strains or consortia, recombinant therapeutic strains, and adjacent bacterial oncology or gene-delivery platforms differ in composition, route, and function (Table 2). FDA’s 2016 LBP CMC guidance excludes microbial gene-therapy vectors and oncolytic bacteria, and its LBP definition excludes vaccines (FDA (U.S. Food and Drug Administration), 2016).

REBYOTA (fecal microbiota, live-jslm), an unengineered suspension prepared from screened donor fecal material, was FDA approved on 30 November 2022 to prevent recurrent *Clostridioides difficile* infection (rCDI), as a single rectal dose after antibacterial treatment. Its pivotal study randomized and treated 262 adults; the Bayesian integrated analysis estimated 8-week treatment success of 70.6% with REBYOTA and 57.5% with placebo (FDA (U.S. Food and Drug Administration), 2022). VOWST (fecal microbiota spores, live-brpk), an oral preparation of live bacterial spores derived from donor fecal material, was approved on 26 April 2023; 8-week CDI recurrence was 12.4% among 89 recipients and 39.8% among 93 placebo recipients (FDA (U.S. Food and Drug Administration), 2023). Both labels warn of a residual risk of transmitting infectious agents. Their E5 status is specific to the approved products and indications and confers no class-wide approval on recombinant or strain-defined LBPs.

In a randomized phase 2b trial after vaginal metronidazole, LACTIN-V, a defined non-engineered product containing *Lactobacillus crispatus* CTV-05, reduced bacterial-vaginosis recurrence by week 12 from 45% with placebo to 30% (risk ratio 0.66, 95% confidence interval 0.44–0.87; 228 randomized), without an imbalance in local or systemic adverse events (Cohen et al., 2020). MTC01, a cultured 15-strain consortium of non-engineered bacteria, was compared with same-donor fecal microbiota transplantation (FMT) in a safety-focused phase 1b trial. Eighteen participants received low- or high-dose products; no adverse event was judged treatment-related, and 7/9 MTC01 versus 8/9 FMT recipients were recurrence-free at week 8 (Bethlehem et al., 2026). The study supports feasibility and engraftment, but was not powered for efficacy equivalence and also exposed strain-specific manufacturing and storage constraints.

Selected recombinant candidates have achieved human proof of mechanism; none of these had marketing authorization at the August 2026 cutoff. An early phase 1 study administered interleukin-10 (IL-10)-secreting, thymidine-dependent *Lactococcus lactis* (LL-Thy12) to 10 patients with Crohn’s disease; it established feasibility and recovery of the modified organism without a controlled efficacy estimate (Braat et al., 2006). SYNB1020, an engineered EcN strain that converts gut ammonia to arginine, showed dose-dependent exposure and pathway activity in healthy volunteers (Kurtz et al., 2019). Development was discontinued in August 2019 after an interim analysis of a randomized, placebo-controlled phase 1b/2a study in 23 patients with cirrhosis. The strain was well tolerated and raised urinary nitrogen, yet blood ammonia did not fall (Synlogic, Inc, 2019). Oral SYNB1618, a chromosomally engineered EcN strain that consumes phenylalanine, was tested in 56 healthy adults and 14 adults with phenylketonuria (PKU). It produced dose-responsive pathway metabolites; adverse events were mainly mild-to-moderate gastrointestinal events, and the strain cleared within 4 days after dosing (Puurunen et al., 2021). In a subsequent 14-day open-label phase 2 trial evaluating SYNB1618 and SYNB1934, 20 participants enrolled and 15 completed treatment overall. SYNB1934 reduced labeled phenylalanine exposure by 43% from baseline and fasting phenylalanine by 40% (Vockley et al., 2023). The pivotal SYNPHENY-3 study was nevertheless terminated in 2024 because interim data indicated that its primary endpoint was unlikely to be met (ClinicalTrials.gov, 2023).

SYNB1353 and NOV-001 provide E3 evidence on gastrointestinal pharmacodynamics and organism fate. SYNB1353, an engineered EcN strain that degrades methionine, was well tolerated in healthy volunteers undergoing an oral methionine challenge and blunted the plasma methionine response by 26% (Perreault et al., 2024); patients with classical homocystinuria were not studied. The NOV-001 program evaluated the engineered *Phocaeicola vulgatus* strain NB1000S with porphyran, a seaweed polysaccharide supplied to support its intestinal persistence, in the adaptive phase 1/2a NOV-001-CL01 trial. The program demonstrated porphyran-dependent abundance but also documented prolonged recovery, evolutionary escape, and large horizontal-gene-transfer events, while prespecified urine-oxalate analyses were not significant (Whitaker et al., 2025). Controlled patient benefit remains unestablished for both programs.

AG019 is an oral engineered Lactococcus lactis strain delivering human proinsulin and IL-10 for recent-onset type 1 diabetes. A phase 1b/2a program analyzed 24 monotherapy and 18 combination-cohort participants. No serious adverse event, AG019-related discontinuation, or systemic exposure to the organism or expressed products was detected; metabolic and immune findings were exploratory (Mathieu et al., 2024). SYNB1891 belongs to a different category: an engineered EcN strain producing a stimulator of interferon genes (STING) agonist after intratumoral injection. Among 32 phase 1 participants, target engagement and stable disease in four were reported; five cytokine-release-syndrome events included one dose-limiting event, with no related infection (Luke et al., 2023). The registry later listed termination for business reasons (ClinicalTrials.gov, 2019). Its route, oncology setting, and immune activation preclude direct extrapolation to mucosal rLBPs.

The phase 1 topical programs ILP100-Topical and AUP1602-C provide E3 evidence. ILP100-Topical contains *Limosilactobacillus reuteri* engineered to express the chemokine C-X-C motif ligand 12 (CXCL12). A randomized phase 1 trial reported a favorable safety profile without systemic exposure and an exploratory healing signal across 240 induced wounds in 36 healthy volunteers (Öhnstedt et al., 2023). AUP1602-C is a topical *Lactococcus cremoris* construct producing human fibroblast growth factor-2, interleukin-4, and colony-stimulating factor-1; the primary report describes it as a gene therapy medicinal product. Its single-arm phase 1 dose-escalation study enrolled 16 patients with non-healing diabetic foot ulcers. Complete healing in five of six high-dose participants provides an uncontrolled signal, with confirmatory efficacy still unestablished (Schindler et al., 2024). Their delivery principles can be compared, with regulatory classification assessed for each product.

### 7.2 CMC and comparability

Similar viable counts can conceal differences in functional output. A proposed release and stability panel therefore combines identity, viability, purity, genetic stability, and mechanism-linked potency (FDA (U.S. Food and Drug Administration), 2016; EDQM (European Directorate for the Quality of Medicines & HealthCare), 2018). Analytical criteria cover characterization, process validation, lot release, stability testing, and *in vivo* follow-up (Supplementary Table 2). These criteria are product-specific and do not constitute a universal regulatory specification.

Strain characterization includes whole-genome sequencing with orthogonal identification and assessment of the recombinant sequence, insertion site, copy number, residual vector sequence, genotype–phenotype relationship, and relevant virulence or antimicrobial-resistance determinants. Qualification of master and working cell banks and the steps from fermentation through fill and hold forms part of the product-specific CMC strategy (FDA (U.S. Food and Drug Administration), 2016). Multi-strain products additionally require strain-specific counts and ratio specifications. Assay selection and acceptance limits depend on the development stage and mechanism of action.

Microbiological purity is assessed through unintended organisms and specified pathogens because the active organism is intentionally present; conventional sterility is therefore inapplicable as an unqualified product claim (FDA (U.S. Food and Drug Administration), 2016; EDQM (European Directorate for the Quality of Medicines & HealthCare), 2018). The proposed risk assessment covers inflammatory components, bacteriophages where relevant, residual media and antibiotics, and clinically usable antimicrobial rescue. Donor-derived products require a separate source-control strategy, including donor qualification, pathogen screening, and traceability; their residual transmissible-agent risk is reflected in the licensed product labels (FDA (U.S. Food and Drug Administration), 2022, 2023).

Potency testing combines viable count for every active strain with a stability-indicating functional assay, such as substrate consumption, product formation, regulated payload expression, or validated immune activity. The intended formulation and route determine the selection of storage, transport, reconstitution, and in-use stress conditions; the resulting test panel is product-specific. Changes in scale, site, medium, process, formulation, or analytics require a comparability strategy, with nonclinical or clinical bridging when residual uncertainty warrants it. MTC01 illustrates the distinction between small-scale feasibility and the manufacturing evidence needed for commercial development (Bethlehem et al., 2026). Applied by analogy, the International Council for Harmonisation (ICH) Q5C guideline supports a product-specific stability-indicating assay panel, and ICH Q5E provides a risk-based approach to comparability before and after manufacturing changes (ICH (International Council for Harmonisation of Technical Requirements for Pharmaceuticals for Human Use), 1995, 2004). Their formal scope is not rLBP-specific; genomic, viable-dose, phenotypic, and functional evidence must be tailored to living products.

### 7.3 Biocontainment and regulatory boundaries

Combined safeguard performance is assessed under conditions capable of exposing shared failures. Experimental nutrient dependence and kill-switch studies support testing rescue conditions and evolutionary escape within the organisms and environments examined (Mandell et al., 2015; Chan et al., 2016).

The common organism-fate assessment quantifies escape frequency with its denominator and detection limit, genetic and functional stability during serial passage and *in vivo* residence, shedding magnitude and duration, clearance, systemic translocation, horizontal gene transfer, environmental survival, and antimicrobial susceptibility. Its scope depends on the indication, route, immune status, and expected exposure. Evidence from a single safeguard or animal model covers only the tested conditions. Repeated dosing or vulnerable populations also require prespecified stopping rules, rescue measures, waste handling, and follow-up for persistent carriage (Frutos-Grilo et al., 2024).

In the United States, FDA’s non-binding 2016 guidance addresses early-clinical LBP CMC; a complete late-stage or commercial development pathway extends beyond its scope (FDA (U.S. Food and Drug Administration), 2016). In Europe, applicable quality considerations include European Pharmacopoeia monograph 3053 (EDQM (European Directorate for the Quality of Medicines & HealthCare), 2018), while classification determines the wider regulatory route. The European Medicines Agency (EMA) identified gaps in tailored nonclinical evaluation in its 2026 concept paper on microbiome-based medicinal products, but the proposed scope expressly excludes products containing genetically modified microorganisms that are classified as advanced therapy medicinal products (ATMPs) (EMA (European Medicines Agency), 2026). Products containing genetically modified organisms may also require product-specific environmental risk assessment (EMA (European Medicines Agency), 2006). Classification and the applicable evidence package are matters for early authority consultation. Consensus recommendations carry a different authority from regulator-issued requirements.

### 7.4 Clinical pharmacology, ethics, and environmental responsibility

Clinical pharmacology relates administered dose to recovered viable organisms, functional activity, shedding, clearance, and host response. Colonization is neither necessary nor sufficient for efficacy. Trial design therefore begins with a prespecified requirement for transient metabolism, mucosal association, durable engraftment, or tumor localization, evaluated by strain-specific culture, quantitative polymerase chain reaction (PCR), sequencing, or barcoding with stated detection limits. Matched antibiotic preconditioning, bowel preparation, route, and supportive care are needed in randomized controls; later trials require patient-relevant efficacy endpoints.

Informed consent covers the product’s capacity for replication, evolution, and shedding, expected clearance, limitations of withdrawal, and available rescue measures (Woodworth et al., 2017). These properties and the vulnerability of the enrolled population determine the monitoring and follow-up needed. In a distinct donor-derived setting, FDA reported transmission of extended-spectrum beta-lactamase-producing *E. coli* through investigational FMT to two immunocompromised adults, with one death (FDA (U.S. Food and Drug Administration), 2019). The proposed donor-governance framework covers consent, traceability, future testing, incidental findings, and look-back. Microbiome profiles can be re-identifiable, supporting data minimization, controlled access, and explicit discussion of privacy uncertainty (Franzosa et al., 2015). Organism-fate measurements inform environmental and household exposure planning, with longer follow-up where persistent or secondary colonization is plausible.

## 8 Conclusion and future perspectives

Licensed donor-derived products establish a manufacturing and clinical precedent for their specific categories and indications. Recombinant therapeutic strains chiefly have early human evidence on safety, organism fate, and pharmacodynamic activity. The termination of the pivotal SYNB1934 study illustrates the need to test mechanistic activity against a prespecified patient-relevant efficacy endpoint; surrogate validity cannot be assumed.

Measurements of active-payload exposure in the intended compartment, induction across relevant biological variation, and retention of function during manufacture and administration provide a basis for comparing preclinical designs. Larger or additional animal models are informative when they resolve a defined uncertainty.
