# Supplementary material for: Engineered live biotherapeutic products: design decisions, failure modes, and clinical translation
Source: Front Microbiol. 2026 Oct 16;17:2002085. doi: 10.3389/fmicb.2026.2002085 (PMC13720282; doi:10.3389/fmicb.2026.2002085)
Supplement: Supplementary file 1 [file Table_1.docx]

Table S1. Construct-level evidence for representative engineered systems

| **Construct, class and route** | **Input and control** | **Output and release mode** | **Model, comparator and measured result** | **Genetic containment and measured clearance** | **Unresolved issue** | **Source** |
| --- | --- | --- | --- | --- | --- | --- |
| Representative therapeutic and delivery systems | | | | | | |
| Dual-key EcN  Genetic platform; oral | Separate *asd*/*thyA* complementation sustains growth; the LuxI/R quorum-sensing module responds to local autoinducer concentration and activates φX174E-mediated lysis. | Treg-biased IL-2 variant and Amuc_1100; bacterial lysis releases the protein payloads. | DSS colitis in mice; PBS, lysis-only and single-plasmid functional-strain controls. Dual-plasmid construct improved inflammatory, immune and barrier readouts. | Genetic design: Δ*asd*/Δ*thyA*, separate complementation and programmed lysis.  Measured fate: blood cultures assessed; quantitative result/LOD NE; post-dose clearance NE. | Effects of autoinducer transport and turnover, nutritional rescue, escape and shedding, and lysis-associated inflammation. Disease specificity requires assessment alongside quorum activation. | Ding et al., 2026 |
| Modified-T3SS engineered *E. coli*  Genetic secretion platform; gut delivery | Engineered type III secretion machinery; operating induction conditions NE. | Functional nanobodies exported through modified T3SS; non-lytic secretion. | Murine gut colonization and secretion-system activity; EcN comparison. Nanobody secretion and binding/function assessed in platform assays; quantitative gut payload exposure NE. | Genetic design: independent containment NE.  Measured fate: gut colonization followed; post-dose clearance and escape frequency NE. | In situ protein concentration, secretion stability and environmental persistence. The genotype described for the Ding EcN construct belongs to a separate platform. | Lynch et al., 2023 |
| EcN-HT  Genetic metabolic platform; oral | Anoxia-linked engineering enables intestinal tyrosine metabolism. | Luminal tyrosine consumption with L-DOPA formation; metabolic substrate processing. | FAH-deficient mice; comparator details NE. Oral treatment lowered tyrosine/toxic metabolite burden and protected against liver injury. | Genetic design: independent containment NE.  Measured fate: organism-distribution and clearance details NE. | Diet/NTBC effects on metabolic flux; L-DOPA fate, construct stability and persistence after dosing. | Gu et al., 2024 |
| e-EcN-HT  Genetic metabolic platform; oral | Engineered intestinal tyrosine metabolism under anoxic conditions. | Luminal tyrosine catabolism; metabolic activity tracked with labeled substrate in a separate species experiment. | FAH-deficient mice/rabbits: disease outcomes. Bama minipigs: ¹³C-tyrosine metabolism, not an FAH-deficient disease model. Comparator details NE. | Genetic design: independent containment NE.  Measured fate: systemic dissemination not detected in reported preclinical assessments; assay matrix/time/LOD NE; post-dose clearance NE. | Diet/NTBC dependence, metabolite fate and durable control. Interpret efficacy and metabolic evidence within the species and model studied. | Gu et al., 2026a |
| Pathogen-sensing EcN  Genetic antimicrobial platform; gut delivery | Senses *P. aeruginosa*-associated signal and activates antimicrobial/antibiofilm functions. | Pathogen-directed antimicrobial and antibiofilm activity; detailed effector/release implementation NE here. | Nematode and mouse gut infection models; wild-type EcN and untreated/control constructs. Pathogen control and infection-related outcomes improved in tested settings. | Genetic design: independent containment NE.  Measured fate: pathogen burden assessed; therapeutic-strain post-dose clearance NE. | Target range, resistance, microbiome effects and shedding; assess persistence of the engineered strain separately from pathogen clearance. | Hwang et al., 2017 |
| AvCystatin-EcN microspheres  Genetic–material platform; oral | Inflammatory biomarker reaches encapsulated bacteria; polyserine-modified alginate plus HA/EGCG provides protection and adhesion. | AvCystatin production/release across the capsule; detailed export pathway NE. | Mouse IBD models; comparator details NE. Improved gastric survival and intestinal adhesion; detectable residence up to 24 h with therapeutic benefit. | Genetic design: independent containment NE.  Measured fate: intestinal residence tracked up to 24 h; viable escape and post-dose clearance NE. | Capsule integrity, sensing kinetics and shedding. Durable engraftment and containment require evidence beyond the observed transient residence. | Zou et al., 2025 |
| Exendin-4 microcapsule depot  Genetic–material platform; subcutaneous | Engineered bacterial production–lysis circuit operates within implanted hydrogel microcapsules. | Bacterial lysis releases exendin-4; the microcapsule is the surrounding depot. | Diabetic rats; comparator details NE. Sustained exendin-4 delivery for approximately 2 weeks and reduced blood glucose. | Genetic design: programmed bacterial lysis; independent containment NE.  Measured fate: live-cell escape and post-dose clearance NE. | Depot durability, output consistency and viable-cell escape. Exclude outcomes from the separate vaccine configuration. | Han et al., 2022 |
| AS1411-EcN-CXCL9  Genetic–material oncology platform; intravenous | AS1411 surface targeting supports tumor accumulation; CXCL9 expression; antibiotics modulate bacterial burden. | CXCL9 delivery from tumor-associated bacteria; output is distinct from the surface-targeting step. | Lewis lung tumor mice; unmodified EcN/PBS and study-specific treatment controls. Increased tumor accumulation, CD8⁺ infiltration and tumor suppression. | Genetic design: independent containment NE.  Measured fate: organ/tumor imaging and CFU measured; values NE. Antibiotics reduced burden while retaining a tumor reservoir; clearance NE. | Circulating and tumor CXCL9 exposure require measurement alongside bacterial distribution; viable organisms may remain after antibiotic debulking. | Gu et al., 2026b |
| Lactate/NIR–ClyA/melittin EcN  Dual-input oncology platform; oral | Lactate regulates ClyA production. NIR generates a photothermal signal that induces melittin. | ClyA accumulates intracellularly; induced melittin drives bacterial lysis and ClyA release. | Colorectal cancer mice; comparator details NE. Dual-responsive system produced antitumor effects; separate input/output quantification NE. | Genetic design: induced lysis module; independent containment NE.  Measured fate: post-dose viable-bacterial clearance NE. | Basal output, induction threshold, thermal exposure and escape. Extraction of a strict Boolean AND truth table remains incomplete. | Chen et al., 2025 |
| **Complementary** control and vesicle **systems** | | | | | | |
| EcN-GadABC@Fe-NE  Genetic–material platform; oral | AMF heats Fe₃O₄, activating the thermal promoter; norepinephrine-derived coating supports delivery. | GadABC-mediated GABA production and release; magnetic heating controls expression. | Restraint-stress mice; PBS and EcN-GFP@Fe-NE controls under AMF. Anxiety-related behavior improved; chemogenetic vagal reactivation attenuated the effect. | Genetic design: independent containment NE.  Measured fate: intestinal retention imaged through 24 h; post-dose clearance NE. | Heating reproducibility, delivered GABA exposure and persistence. Organism clearance requires assessment after expression shuts off. | Wang et al., 2025  . |
| EcN-pDawn-φX174E/TRAIL  Genetic oncology platform; systemic delivery (route details NE) | Blue-light-responsive pDawn activates φX174E-mediated lysis; NIR fluorescence supports imaging. | Bacterial lysis releases TRAIL. | Mouse tumor model; illuminated versus non-illuminated construct. Tumor-growth inhibition: 53% with light versus 11.8% without light. | Genetic design: light-triggered lysis; independent containment NE.  Measured fate: post-treatment viable-bacterial clearance NE. | Illumination coverage, lysis escape and endotoxin burden; blue-light treatment and NIR imaging remain separate functions. | Zhang et al., 2022 |
| EcN-*flaB*–UCNP  Genetic–material oncology platform; intravenous | 808-nm NIR is converted to blue light by UCNPs; photoactivated EL222 drives pC120-controlled *flaB* expression in bacteria. | Bacteria produce and secrete flagellin B; the payload engages host-cell TLR5 and initiates MyD88-dependent innate immune signaling. | Mouse tumor studies; comparator details NE. Antitumor immune responses and tumor control observed. | Genetic design: independent containment NE.  Measured fate: organism-distribution and post-dose clearance details NE. | Local photon delivery, basal expression, particle fate and viable-bacterial persistence; exposure dosimetry requires information beyond wavelength. | Zhu et al., 2023 |
| MG1655-pDawn-HlyE@UCNP  Genetic–material oncology platform; intravenous | NIR-to-blue upconversion activates pDawn-controlled expression. | HlyE generation and secretion from engineered bacteria. | Mouse tumor studies; comparator details NE. NIR-activated system suppressed tumor growth. | Genetic design: independent containment NE.  Measured fate: organism-distribution and post-dose clearance details NE. | Tumor photon exposure, basal toxin output, nanoparticle persistence and rescue susceptibility. | Tao et al., 2023 |
| MG1655 Ec@DIG-GVs  Genetic–material oncology platform; intratumoral and intravenous experiments | Gas vesicles support ultrasound imaging; hHIFU heating activates IFN-γ expression; acidic conditions release conjugated DOX. | IFN-γ production/secretion plus DOX release; imaging and treatment are distinct functions. | 4T1 tumor mice; matched ±hHIFU and component controls. Tumor and immune readouts improved; routes evaluated in separate experiments. | Genetic design: independent containment NE.  Measured fate: organ/tumor bacterial CFU after intravenous dosing measured; quantitative values and terminal clearance NE. | Heating accuracy, IFN-γ/DOX exposure and viable-organism persistence. Clearance assessment requires longitudinal biodistribution measurements. | Yang et al., 2024 |
| MG1655 PD-1@EcM  Genetic oncology platform; intravenous after thermal ablation | Acid-responsive promoter controls mouse anti-PD-1 scFv expression. | Anti-PD-1 scFv production; extracellular release/availability NE. | Post-ablation hepatocellular carcinoma mice; single administration; comparator details NE. CD8⁺/macrophage changes, tumor control and survival benefit reported. | Genetic design: independent containment NE.  Measured fate: post-dose viable-bacterial clearance NE. | Extracellular functional scFv exposure, acid selectivity and bacterial persistence; verify checkpoint blockade at the target alongside expression. | Li et al., 2025 |
| EcN Δ*nlpI*/OMV–LOx (ENHL)  Genetic–vesicle oncology platform; oral | Δ*nlpI* increases OMV production; arabinose induces the LOx-bearing delivery system. | Bacteria generate LOx-bearing OMVs for lactate depletion and radiotherapy sensitization. | Orthotopic colorectal cancer mice; PBS, control strain, ENHL and radiation/component controls. Tumor lactate decreased and radiotherapy response improved. | Genetic design: OMV enhancement and inducible expression; independent containment NE.  Measured fate: tumor bacterial burden measured; post-dose clearance NE. | Inducer exposure, cargo heterogeneity, live-cell versus OMV and payload-specific contributions, vesicle transport and bacterial shedding. Matched controls should resolve effects of immunostimulatory lipopolysaccharide and membrane proteins. | Peng et al., 2026 |

**Notes.** The 16 records are a non-exhaustive selection of preclinical constructs. Four records (Ding et al., 2026; Gu et al., 2024; Gu et al., 2026a; Hwang et al., 2017) correspond to the three intestinal strategies in Figure 2. Ding et al. is recorded once; Gu 2024 and 2026a describe separate constructs/studies.

Class labels describe the engineering approach; regulatory classification requires separate assessment. All model outcomes in this table are preclinical; routes, species, controls and endpoints remain study-specific.

Genetic design, measured organism fate and post-dose clearance are recorded separately. For systems using programmed lysis, auxotrophy, material encapsulation, expression shutoff or antibiotic debulking, complete containment and clearance require direct evidence.

NE, not extracted: a field or detailed original-paper location has not been fully extracted. Reporting status remains unresolved; NE is distinct from NR (not reported). Not detected denotes a reported assay result, with unextracted sampling conditions or detection limits recorded as NE.

Original-paper and review locations are listed separately. Abstract denotes abstract-level support; unextracted panels are marked NE. Unresolved issues are validation questions proposed by the review authors, not a complete assessment of the original study.

AMF, alternating magnetic field; Amuc_1100, an *Akkermansia muciniphila*-derived membrane protein; AvCystatin, a cysteine-protease inhibitor from Acanthocheilonema viteae; CFU, colony-forming units; ClyA, cytolysin A; CXCL9, C-X-C motif chemokine ligand 9; DOX, doxorubicin; DSS, dextran sulfate sodium; EcN, *Escherichia coli* Nissle 1917; EL222, blue-light-responsive transcription factor used with the pC120 promoter; FAH, fumarylacetoacetate hydrolase; *flaB*, flagellin B gene; GABA, γ-aminobutyric acid; GFP, green fluorescent protein; HA/EGCG, hyaluronic acid/epigallocatechin gallate; hHIFU, hyperthermic high-intensity focused ultrasound; HlyE, hemolysin E; IBD, inflammatory bowel disease; IFN-γ, interferon-γ; IL-2, interleukin-2; L-DOPA, L-3,4-dihydroxyphenylalanine; LOD, limit of detection; LOx, lactate oxidase; LuxI/R, LuxI/LuxR quorum-sensing module; MyD88, myeloid differentiation primary response 88; NIR, near-infrared; NTBC, nitisinone; OMV, outer membrane vesicle; PBS, phosphate-buffered saline; PD-1, programmed cell death protein 1; scFv, single-chain variable fragment; T3SS, type III secretion system; TLR5, Toll-like receptor 5; TRAIL, tumor necrosis factor-related apoptosis-inducing ligand; Treg, regulatory T cell; UCNP, upconversion nanoparticle. *asd* and *thyA* denote the auxotrophy-related genes deleted in the dual-key EcN construct; φX174E denotes the bacteriophage-derived lysis gene.

Table S2. Quality, functional stability and organism-fate assessment across development stages

| **Attribute** | **Assay or challenge** | **Readout and method limits** | **Development stage and route** | **Interpretation boundary** | **Source and basis** |
| --- | --- | --- | --- | --- | --- |
| **Identity** | | | | | |
| Strain identity | Whole-genome sequencing against a qualified strain reference. | Genome identity, coverage and variant calls; define variant-detection sensitivity. | Characterization; cell-bank qualification; changes affecting identity. | Therapeutic-strain identification requires resolution beyond the species level; WGS is proposed here as one method. | (FDA, 2016)  Guidance + synthesis |
| Orthogonal identity | Independent biochemical, phenotypic or targeted genetic identification. | Concordance with the qualified reference; method-specific acceptance criteria. | Characterization; lot release if justified. | Use complementary evidence to detect substitution or mix-up; avoid treating repeated measurements of one target as independent proof. | (FDA, 2016)  Guidance + synthesis |
| Construct integrity | Verify insert sequence, locus, copy number and unintended vector sequence. | Sequence concordance; copy number per genome; detection sensitivity for unintended sequence. | Characterization; process validation; stability. | Link genotype to measured phenotype and therapeutic activity. | (FDA, 2016)  Guidance + synthesis |
| Retained plasmid stability | Passage without artificial selection where relevant; test process and storage recovery. | Plasmid-positive fraction, denominator, generations and corresponding activity. | Characterization; process validation; stability. | Plasmid maintenance is distinct from organism containment; retention without function is insufficient. | (FDA, 2016; EDQM, 2018)  Guidance / pharmacopoeial context + synthesis |
| **Purity** | | | | | |
| Unintended organisms | Product-specific culture or molecular assays for contaminants and specified pathogens. | Counts per defined mass or volume; method recovery, LOD and specified-organism result. | Process validation; lot release where justified. | An intentionally live product cannot be assessed by an unqualified conventional sterility claim. | (FDA, 2016; EDQM, 2018)  Guidance / pharmacopoeial context + synthesis |
| Inflammatory components | Route- and dose-matched evaluation of endotoxin or other relevant bacterial components. | Assay activity per dose or sample amount; interference controls and justified limits. | Characterization; risk-based lot release. | Lysis can change inflammatory exposure. No universal numerical limit is proposed for all strains and routes. | (FDA, 2016)  Guidance context + synthesis |
| Process residues | Measure relevant media components, antibiotics and processing aids after clearance steps. | Residual mass or concentration per dose; recovery, LOD or LOQ as appropriate. | Process validation; lot release if justified. | Residual-antibiotic analysis and susceptibility of the live strain address different questions. | (FDA, 2016)  Guidance + synthesis |
| Relevant bacteriophages | Qualified phage-detection assay appropriate to strain, raw materials and process. | Detected target or infectivity; sample amount and sensitivity of the selected method. | Cell-bank qualification; process validation; lot release if justified. | A negative assay excludes detectable targets only within its specified conditions and scope. | (FDA, 2016)  Guidance context + synthesis |
| **Dose and function** | | | | | |
| Strain-specific viable dose | Validated enumeration of each active strain; distinguish members of mixed products. | CFU or justified viable-cell measure per dose; strain ratios; recovery conditions. | Lot release; stability. | Viable dose and functional potency are separate attributes; pooled counts can conceal ratio drift. | (FDA, 2016)  Guidance + synthesis |
| Mechanism-linked potency | Stability-indicating assay of substrate consumption, product formation or validated biological activity. | Activity per dose or time in a defined matrix; assay range and suitable reference. | Characterization; lot release; stability. | Select a mechanism-specific functional assay to complement CFU measurements. | (FDA, 2016; ICH, 1995)  Guidance / analogy + synthesis |
| Basal and induced output | No-input, relevant-input and off-target conditions; activation and recovery series. | Basal and induced activity separately; ratio only with a valid baseline; kinetics and threshold. | Characterization; lot release if justified; stability. | Maximum induction alone omits basal leakage and reversibility; conditions must be physiologically relevant. | (Ozdemir et al., 2018)  Synthesis informed by review |
| Activity per viable cell | Pair functional assay with viable enumeration on comparable samples. | Activity per CFU or justified viable-cell denominator, with sampling time and recovery method. | Characterization; lot release if justified; stability. | Normalize only when denominator and cell state are comparable. Similar counts can conceal functional drift. | (Charbonneau et al., 2020)  Synthesis informed by review |
| **Stability** | | | | | |
| Viable dose over shelf life | Test release and justified storage time points through shelf-life end. | Viable amount per dose at each time; storage and recovery conditions. | Lot release; stability. | Assess viability at release and at the shelf-life endpoint, with functional activity tracked in parallel. | (FDA, 2016; ICH, 1995)  Guidance / analogy + synthesis |
| Construct and function retention | Sequence and phenotype after storage recovery or relevant passage. | Producer fraction, construct retention and activity per viable cell; generations or storage time. | Characterization; stability. | Population averages or one endpoint sequence can miss minority non-producing variants. | (Leeflang et al., 2025; Rugbjerg et al., 2018)  Synthesis / study evidence |
| Preservation stress | Relevant freeze–thaw, lyophilization, oxygen and moisture challenges. | Challenge duration and intensity; viable recovery and retained functional activity. | Process validation; stability. | Count-based interpretation depends on recovery conditions; consider viable but nonculturable cells where relevant. | (EDQM, 2018; ICH, 1995)  Pharmacopoeial context / analogy + synthesis |
| Handling and route barriers | Transport, reconstitution and in-use holding; gastrointestinal challenge for oral products. | Time, temperature and matrix; viable dose and activity after each relevant exposure. | Process validation; stability; route characterization. | Assess in vivo survival and target-site function alongside simulated-fluid survival. Select conditions for the actual route. | (FDA, 2016; Heavey et al., 2022)  Guidance context + synthesis |
| **Manufacture** | | | | | |
| Culture and passage control | Qualify fermentation, medium and passage limits over the proposed process. | Defined process ranges, generations and associated dose, potency and stability. | Process validation. | Peak yield alone can obscure selection for non-producing cells; justify the generation limit. | (FDA, 2016)  Guidance + synthesis |
| Downstream process control | Evaluate harvest, washing, formulation, preservation, filling and hold steps. | Step-specific parameters and hold times; viable recovery, functional retention and relevant residues. | Process validation. | Define controls for critical steps and select the development assays appropriate for routine lot testing. | (FDA, 2016; EDQM, 2018)  Guidance / pharmacopoeial context + synthesis |
| Master and working cell banks | Qualify identity, purity, viability, phenotype, genetic stability and adventitious agents. | Bank-specific test results, passage history and method sensitivity where applicable. | Characterization; cell-bank qualification. | Bank qualification supports process consistency but cannot replace finished-product dose and potency testing. | (FDA, 2016)  Guidance + synthesis |
| Change comparability | Compare representative lots before and after scale, site, process, formulation or analytical changes. | Differences in identity, phenotype, viable dose, function and stability; justified bridging. | Characterization; process validation. | Matching CFU alone is insufficient. Additional nonclinical or clinical evidence depends on unresolved uncertainty. | (ICH, 2004)  Analogy + synthesis |
| **Organism and DNA fate** | | | | | |
| Escape and nutrient rescue | Challenge safeguards in relevant host and environmental matrices; test rescue and stability. | Escape events / total challenged population; conditions, time and detection bound. | Characterization; containment stability. | Nutrient rescue or ecological context can change apparent control. The denominator and recovery conditions are essential. | (Rottinghaus et al., 2022)  Study evidence + synthesis |
| Post-dose shedding and clearance | Serial, strain-specific culture with complementary molecular identification where appropriate. | Viable amount per specimen; sampling schedule, follow-up and LOD. | In vivo follow-up; route-specific specimens. | Eradication assessment requires follow-up beyond a single negative sample; inferring viable persistence from molecular detection requires complementary viability evidence. | (Kurtz et al., 2019; Puurunen et al., 2021)  Study evidence + synthesis |
| Non-target distribution | Quantify viable bacteria in target and non-target compartments; add molecular assays as needed. | CFU per tissue mass or specimen; site, time, recovery efficiency and LOD. | Characterization; in vivo follow-up, matched to route. | Confirm viable translocation with evidence complementary to fluorescence or DNA detection, and measure payload exposure separately from bacterial distribution. | (Gu et al., 2026b)  Study evidence + synthesis |
| Engineered DNA and transfer | Track engineered sequence persistence; use recipient-linked evidence to test transfer. | DNA amount or detected fraction; recipient identity, transfer denominator and method sensitivity. | Characterization; relevant in vivo or environmental follow-up. | Separate residual DNA, recipient association and demonstrated transfer. Assess DNA persistence after loss of viable producer cells. | (Whitaker et al., 2025)  Study evidence + synthesis |

**Notes.** Assays, units, denominators, acceptance limits and detection limits require product- and method-specific justification. Characterization, process validation, lot release, stability testing and in vivo follow-up serve different purposes; routine release panels use a selected subset.

Basis labels distinguish guidance, pharmacopoeial context, analogy, study evidence and author synthesis. Citations provide context or support for each attribute; the row-specific methods, interpretive limits and stage assignments reflect author synthesis. FDA (2016) addresses early-clinical LBP CMC. ICH Q5C and Q5E provide analogies for rLBP development, subject to their formal scopes. EDQM (2018) is the cited notice on pharmacopoeial quality requirements; numerical acceptance limits require separate justification.

Study examples support the relevant attribute; the cited report defines the assays and follow-up performed.

CFU, colony-forming units; CMC, chemistry, manufacturing and controls; DNA, deoxyribonucleic acid; EDQM, European Directorate for the Quality of Medicines & HealthCare; FDA, US Food and Drug Administration; ICH, International Council for Harmonisation; LBP, live biotherapeutic product; LOD, limit of detection; LOQ, limit of quantification; rLBP, recombinant LBP; WGS, whole-genome sequencing.
